# Microbial biomass in compost during colonization of *Agaricus bisporus*

**DOI:** 10.1186/s13568-016-0304-y

**Published:** 2017-01-03

**Authors:** Aurin M. Vos, Amber Heijboer, Henricus T. S. Boschker, Barbara Bonnet, Luis G. Lugones, Han A. B. Wösten

**Affiliations:** 1 Department of Microbiology, Department of Biology, Utrecht University, Padualaan 8, 3584 CH Utrecht, The Netherlands; 2 Biometris, Wageningen University, Droevendaalsesteeg 1, 6708 PB Wageningen, The Netherlands; 3 Koninklijk Nederlands Instituut voor Onderzoek der Zee, Korringaweg 7, 4401 NT Yerseke, The Netherlands; 4 Somycel S.A-Sylvan, Z.I. Sud, Route de Tours, 37130 Langeais, France

**Keywords:** Fungi, Basidiomycete, *Agaricus bisporus*, Mushroom, Compost, Microbial biomass

## Abstract

*Agaricus bisporus* mushrooms are commercially produced on a microbe rich compost. Here, fungal and bacterial biomass was quantified in compost with and without colonization by *A. bisporus*. Chitin content, indicative of total fungal biomass, increased during a 26-day period from 576 to 779 nmol *N*-acetylglucosamine g^−1^ compost in the absence of *A. bisporus* (negative control). A similar increase was found in the presence of this mushroom forming fungus. The fungal phospholipid-derived fatty acid (PLFA) marker C18:2ω6, indicative of the living fraction of the fungal biomass, decreased from 575 to 280 nmol g^−1^ compost in the negative control. In contrast, it increased to 1200 nmol g^−1^ compost in the presence of *A. bisporus*. Laccase activity was absent throughout culturing in the negative control, while it correlated with the fungal PLFA marker in the presence of *A. bisporus.* PLFA was also used to quantify living bacterial biomass. In the negative control, the bacterial markers remained constant at 3000–3200 nmol PLFA g^−1^ compost. In contrast, they decreased to 850 nmol g^−1^ compost during vegetative growth of *A. bisporus*, implying that bacterial biomass decreased from 17.7 to 4.7 mg g^−1^ compost. The relative amount of the Gram positive associated PLFA markers a15:0 and a17:0 and the Gram negative PLFA associated markers cy17:0 and cy19:0 increased and decreased, respectively, suggesting that Gram negative bacteria are more suppressed by *A. bisporus.* Together, these data indicate that fungal biomass can make up 6.8% of the compost after *A. bisporus* colonization, 57% of which being dead. Moreover, results show that *A. bisporus* impacts biomass and composition of bacteria in compost.

**Electronic supplementary material:**

The online version of this article (doi:10.1186/s13568-016-0304-y) contains supplementary material, which is available to authorized users.

## Introduction

Compost is used for the commercial production of the fruiting bodies of *Agaricus bisporus* known as button mushrooms. In the Netherlands, compost is produced from a mixture of wheat straw, horse manure, gypsum, and water, while chicken manure is either or not used as an additional nitrogen source (Gerrits 1988). Phase I (PI) of composting takes 3–6 days during which temperature increases to 80 °C due to microbial activity. Temperature of the compost during phase II (PII) is initially 50 °C, followed by a 2-day-period at 60 °C, and a 3-day-period at 45 °C (Gerrits 1988). The thermophilic fungus *Scytalidium thermophilum* (also known as *Humicola insolens*; Straatsma and Samson 1993) removes ammonia during PII and suppresses competitors of *A. bisporus* (Ross and Harris 1983; Straatsma et al. 1989, 1994). At the end of PII, 50–60% of xylan and cellulose have been degraded as compared to the starting material, while lignin is still largely intact (Jurak 2015; Jurak et al. 2015). Introduction of *A. bisporus* spawn in PII-end compost initiates phase III (PIII). This phase takes 16–19 days, during which the button mushroom colonizes the compost at 25 °C. This is accompanied by degradation of 50% of the lignin, with an additional decomposition of 15% of the xylan and 10% of the cellulose (Jurak 2015; Jurak et al. 2015). PIII-end compost is either or not supplemented with formaldehyde treated, protein rich nutrients (Gerrits 1988), after which Phase IV (PIV) is initiated by topping the compost with a casing layer that consists of peat and lime. Casing is essential for mushroom formation (Visscher 1988), probably due to bacterial activity that removes suppressing volatiles produced by *A. bisporus* (Noble et al. 2009). CO_2_ levels are high and relative humidity is 85% during colonization of the casing layer. Mushroom formation is induced after 7 days by lowering the compost temperature to 18–22 °C, increasing relative humidity up to 90%, and lowering CO_2_ levels by venting (Visscher 1988). Mushrooms are harvested in 2–3 flushes with 7–8 day intervals with a typical yield of 30 kg m^−2^ and a bulk density of 85–95 kg m^−2^. A total of 44, 29, and 8% of cellulose, xylan, and lignin is degraded in PIV, respectively, when compared to PIII-end. Thus, a significant amount of the organic compounds remain unused in the compost after cultivation of button mushrooms (Iiyama et al. 1994; Chen et al. 2000; Jurak 2015). It should be noted that cellulose has been quantified based on glucose content in the compost. Since glucan in the fungal cell wall also consists of glucose, total cellulose degradation in PI–PIV will be higher than that reported.

Wheat straw represents the major carbon source for *A. bisporus* in compost but microbes may also represent an important part of its diet (Sparling et al. 1982). So far, dynamics of microbial biomass in compost has not been monitored. Here, we determined fungal and bacterial biomass in compost in time in the presence and absence of *A. bisporus* using chitin, laccase, and phospholipid-derived fatty acid (PLFA) analysis. Quantification of chitin determines both living and dead mycelium (Ekblad et al. 1998), while laccase activity can be used to monitor the linear vegetative growth phase (Wood 1979; Wood and Goodenough 1977). PLFA assays are used to determine the fraction of both living fungal and bacterial biomass (Frostegård et al. 2011). Experimental data indicate that *A. bisporus* can make up 6.8% of the dry weight of compost, more than half of it being dead after 26 days of growth. In addition, experimental data show that *A. bisporus* suppresses growth of bacteria and impacts the bacterial composition.

## Materials and methods

### Strains and growth conditions

The *A. bisporus* strain A15 (Sylvan, Horst, the Netherlands) was routinely grown at 25 °C on malt extract agar (MEA; 20 g l^−1^ malt extract agar [BD biosciences, Franklin Lakes, USA], 2.1 g l^−1^ MOPS, pH 7.0, and 1.5% agar). Spawn was made by mixing pieces of colonized MEA (0.5 by 0.5 cm) with a sterilized mixture of 50 g rye, 1.4 g CaCO_3_, 1 g CaSO_4_, and 50 ml demi water. The rye was colonized in 3 weeks at 25 °C and stored at 4 °C before use. PII-end compost (CNC Grondstoffen, Milsbeek, The Netherlands) was inoculated (5 spawn grains per 25 g of compost) in 250 ml bottles and incubated in the dark at 25 °C for up to 26 days. Compost not inoculated with *A. bisporus* served as a control. Samples of PII-end compost and PIII compost were stored at −20 °C.

Compost agar medium (CAM) was prepared by homogenizing 75 g phase III compost (CNC Grondstoffen) in 0.5 l water. The mixture was autoclaved three times at 120 °C for 20 min and mixed 1: 1 with 3% agar. To produce pure *A. bisporus* mycelium CAM was overlaid with a polycarbonate (PC) membrane (diameter, 76 mm; pore size, 0.1 µm; Profiltra, Almere, the Netherlands) and inoculated at the center of the plate. After 14 days at 25 °C mycelium was lyophilized, and ground to a powder.

### Laccase activity

Compost extract was prepared by mixing 50 mg lyophilized and milled compost with 1 ml demi water and incubating head over tail for 1 h at 25 °C. Insolubles were removed by centrifugation at 4 °C and 15,000*g* for 15 min. Laccase activity was determined by adding 20 µl five times diluted compost extract with 1 ml 1 mM ABTS in citric phosphate buffer, pH 4. Change in absorbance was followed at 420 nm for 30 s. Activity in units (U) was calculated using the law of Lambert–Beer with an extinction factor of 36,000 M^−1^ cm^−1^.

### Quantification of chitin

*N*-Acetylglucosamine (GlcNAc) released from chitin was quantified in technical triplicates using a colorimetric assay (Reissig et al. 1955; François 2006). Compost (20–30 mg dry weight) was mixed with 1 ml 6% KOH (w/v) and incubated at 80 °C for 90 min. The mixture was centrifuged at 10,000*g* at 4 °C for 10 min after adding 500 µl glacial acetic acid. Pellets were washed twice with water and once with 50 mM potassium phosphate buffer, pH 6.5. Chitin was digested overnight in 600 µl 50 mM potassium phosphate buffer pH 6.5 containing 0.42 U chitinase, 8.3 U lyticase, and 2 µl protease inhibitor (P8215, Sigma-Aldrich, St Louis, USA). Water was added to a final volume of 1.2 ml and 100 µl was used for colorimetric quantification of GlcNAc. To this end, 50 µl demi water and 150 µl 0.27 M tetraborate were added. After incubation at 100 °C for 15 min, 1.8 ml Reissig reagent (10 g 4-(dimethylamino)benzaldehyde, 12.5 ml of 10 M HCl, and 87.5 ml glacial acetic acid) was added and incubated at 40 °C for 20 min. Liquid was transferred to a cuvette and the OD_585_ was measured. A standard curve of 0–90 nmol GlcNAc (with a detection limit of 2 nmol) was used to calculate the release of the aminosugar per gram compost after subtracting the signal obtained from the assay in the absence of chitinase. 0.5–8 mg of pure mycelium was used to determine the amount of GlcNAc release mg^−1^ mycelium. The average OD of the reaction mixture in the absence of compost was substracted from the OD of mixtures containing fungal colonized compost or pure mycelium. GlcNAc was not detected when using 3 × 10^9^ dH5α *Escherichia coli* cells that contain a total amount of 174 nmol of the aminosugar (Wientjes et al. 1991). This shows that the GlcNAc in the assay does not orginate from bacterial biomass.

### Phospholipid fatty acid analysis

PLFAs were extracted from 300 mg compost as described (Frostegård et al. 1991, 1993; Hedlund 2002) based on the methods of Bligh and Dyer (1959) and White et al. (1979). In short, PLFAs were extracted using 10 ml Bligh and Dyer solution (CHCl_3:_MeOH:citrate buffer, 1:2:0.8 v/v/v). Phases were separated by addition of 4 ml CHCl_3_ and 4 ml citrate buffer during overnight incubation at room temperature. An aliquot of 3 ml of the lipid extract was transferred to a glass tube. After evaporation of the solvent with a stream of N_2_, samples were solubilized in CHCl_3_ and applied on a silica column. Phospholipids were eluted with 1.5 ml MeOH after eluting the neutral and glycolipids with 1.5 ml CHCl_3_ and 6 ml acetone, respectively. 5 µg of methyl nonadecanoate (C19:0, Sigma-Aldrich) was added to each sample as an internal standard. Samples were transesterified at 37 °C for 15 min by addition of 1 ml toluene:methanol (1:1) and 1 ml freshly made 0.2 M KOH in methanol. After cooling to room temperature for 20 min, 2 ml hexane:CHCl_3_ (4:1 v/v), 0.3 ml 1 M HAc, and 2 ml H_2_O were added. Samples were centrifuged for 5 min at 685 g and 5 µg of methyl dodecanoate (C12:0, Sigma Aldrich) was added as an internal control to the upper phase that contained the PLFAs. After evaporation of the solvent by a stream of N_2_, lipids were taken up in 200 µl hexane for gas chromatography-flame ionization detector analysis. PLFAs were identified based on retention time and equivalent chain length as calculated using C12:0, C16:0, and C19:0. Abundance in nmol g^−1^ was calculated using spiked C19:0. The C18:2ω6 marker was used to estimate fungal biomass, while various PLFA markers (Additional file 1: Table S1) were used as markers of bacterial biomass (Frostegård and Bååth 1996; Hedlund 2002; Ruess and Chamberlain 2010).

### Data analysis

Custom R (v3.03) scripts were used for annotation of PLFAs. Statistical analysis of chitin and PLFA biomass was done with t tests in SPSS Statistics 22 software. Changes of laccase, chitin, and PLFA over time were analysed using ANOVA with Bonferroni or Dunnet T3 post hoc correction in SPSS Statistics 22 software. In all cases p was <0.05.

## Results

### Fungal biomass in compost

**Fig. 1:**
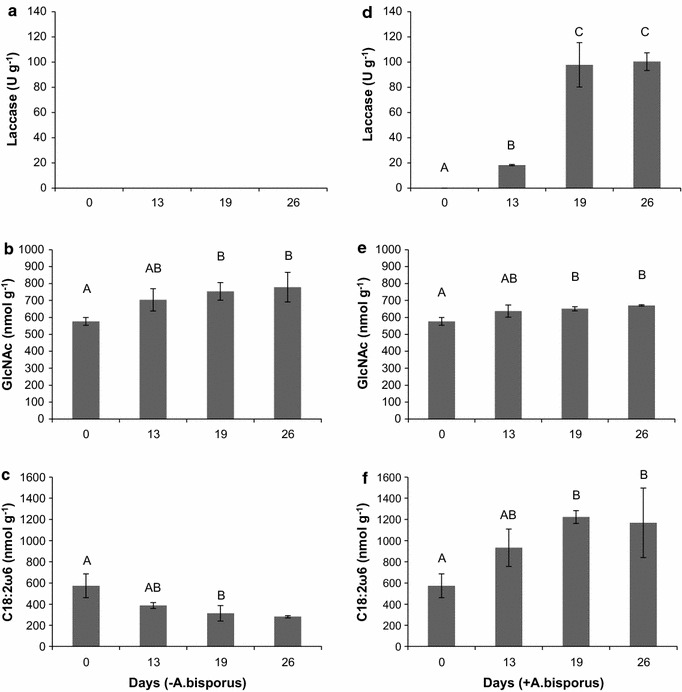
Fungal biomass in compost. Fungal biomass as determined by laccase activity (U g^−1^) (**a**, **d**) and chitin (GlcNac, nmol g^−1^) (**b**, **e**) and PLFA (C18:2ω6, nmol g^−1^) (**c**, **f**) content in compost that had not (**a**, **b**, **c**) or had (**d**, **e**, **f**) been colonized with *A. bisporus*. *Error bars* indicate standard deviation. Significant differences are indicated with *letters* (ANOVA with Bonferonni or Dunnett T3 correction)

*Agaricus bisporus* strain A15 was grown in PII-end compost for 26 days. Fungal biomass was determined by laccase activity and by chitin and PLFA content. No laccase activity was found in the negative control (PII-end compost that had not been inoculated with *A. bisporus*) during the 26 days of incubation (Fig. 1). In contrast, laccase activity increased to approximately 100 U g^−1^ compost after 19–26 days of colonization by *A. bisporus*. Fungal biomass based on chitin content increased during the first 19 days from 576 to 754 nmol GlcNAc g^−1^ in the absence of *A. bisporus*, after which it did not increase further (Fig. 1). In the presence of *A. bisporus*, an increase in the released amino sugar was observed between day 0 and 19 from 576 to 651 nmol GlcNAc g^−1^ compost (Fig. 1), after which no further increase was found. No significant differences in chitin content were observed in compost with or without *A. bisporus* throughout culturing. The amount of the fungal PLFA marker C18:2ω6 decreased during the 26-day-period from 575 to 280 nmol g^−1^ in the absence of *A. bisporus* (Fig. 1). In contrast, it increased from 575 to 1200 nmol during the first 19 days in the presence of *A. bisporus*, after which it remained constant.

Pure *A. bisporus* mycelium grown on compost agar medium contained 40 nmol mg^−1^ C18:2ω6, and 9.9 nmol GlcNAc mg^−1^ (Additional file 1: Figure S1). These amounts were used to calculate the fungal biomass in compost. The estimated biomass in PII-end compost based on chitin and PLFA was 58 and 14 mg g^−1^, respectively (Table 1). These amounts had increased to 68 and 29 mg g^−1^ compost after 26 days of growth of *A. bisporus*.

**Table 1:** Fungal biomass in mg g^−1^ compost based on chitin (GlcNAc release) and PLFA C18:2ω6 content

Methods	Day 0^a^			Day 13			Day 19			Day 26		
	µ	σ		µ	σ		µ	σ		µ	σ	
Chitin	58.1	2.3	A	64.2	0.3	A	65.6	3.2	A	67.6	1.5	A
C18:2ω6	14.2	2.8	B	23.0	4.4	B	30.2	1.5	B	28.9	8.1	B

Average biomass (µ) and standard deviation (σ) are shown. Significant differences between the biomass at each time point are indicated with letters (p < 0.05, t test)

^a^Based on conversion factor of *A. bisporus*

### Bacterial biomass in compost

**Fig. 2:**
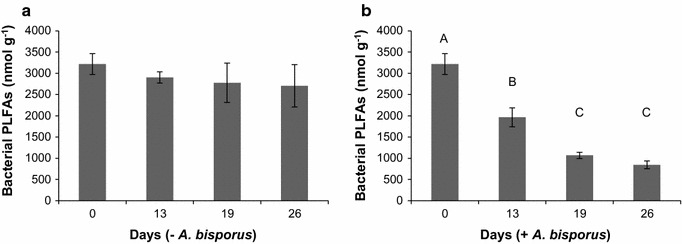
Bacterial biomass in compost based on PLFA. Sum of bacterial PLFAs in nmol g^−1^ compost in the absence (**a**) and presence (**b**) of *A. bisporus*. *Error bars* indicate standard deviation. Statistical differences are indicated with *letters*, absence of letters indicates no significant differences were found (ANOVA, Bonferroni correction)

PLFA was used to quantify bacterial biomass in compost that had either or not been inoculated with *A. bisporus.* To this end, selected bacterial PLFA markers were used (Additional file 1: Table S1). Bacterial biomass remained constant between day 0 and 26 in the absence of *A. bisporus* (Fig. 2a). In contrast, bacterial PLFAs decreased from 3200 to 850 nmol g^−1^ in this time period in the presence of *A. bisporus* (Fig. 2b). Only minor temporal changes in relative abundance of the bacterial markers were found in the absence of *A. bisporus*. In contrast, relative abundance of the bacterial markers a15:0, C15:0, and a17:0 increased 1.3-, 2.8-, and 1.2-fold, respectively, from day 13 onwards, while relative abundance of 16:1ω9, 10Me16:0, cy17:0, and cy19:0 decreased 5-, 1.4-, 2.5-, and 1.8-fold, respectively (Table 2). The contribution of *A. bisporus* PLFAs to the pool of bacterial PLFAs was ≤5% after 26 days of compost colonization. From these data and the assumptions that 363.6 nmol bacterial PLFA equals 1 mg C and bacterial biomass consists for 50% of C (Bratbak and Dundas 1984; Tsiafouli et al. 2015) it is concluded that bacterial biomass decreased from 17.7 to 4.7 mg g^−1^ compost during the 26-day-colonization of compost by *A. bisporus*. In contrast, bacterial biomass was in the range of 14.9–17.7 mg g^−1^ compost in the absence of *A. bisporus*.

**Table 2:** Relative abundance of selected bacterial PLFAs in compost in the absence (−) and presence (+) of *A. bisporus*, and relative abundance of the selected bacterial PLFAs in pure *A. bisporus* mycelium (in mol%)

Days	Selected bacterial PLFAs in mol%									
	i15:0	a15:0	C15:0	i16:0	16:1ω9	10Me16:0	a17:0	cy17:0	C18:1ω7	cy19:0
− *A. bisporus*										
0	17.3	14.7	2.4	23.2	0.8	4.5	9.2	8.6	9.2	10.2
13	18.6	13.9	1.8	20.7	0.9	5.5	7.7	11.1	7.9	11.9
19	19.6	14.7	1.8	20.4	0.9	5.5	7.5	10.9	7.5	11.3
26^a^	18.8	15.0	1.9	20.4	0.9	5.0	7.3	10.6	8.4	11.6
+ *A. bisporus*										
0	17.3	14.7	2.4	23.2	0.8	4.5	9.2	8.6	9.2	10.2
13	17.4	15.5	2.8	22.1	0.5	4.9	10.1	10.2	5.8	10.8
19	15.6	17.9	5.0	23.8	0.4	4.2	12.3	6.0	6.4	8.4
26	16.2	19.6	7.8	24.3	0.1	3.4	12.3	4.0	6.3	6.1
Pure mycelium	11.4	11.2	39.8	14.6	2.4	1.1	0.0	3.6	14.3	1.5

Values are the average of three biological replicas

^a^Average of 2 replicates

## Discussion

Fungal and bacterial biomass was quantified during a 26-day-incubation of PII-end compost in the presence and absence of *A. bisporus.* Chitin was used to quantify total fungal biomass, laccase and C18:2ω6 for the living fraction of the fungal biomass, and bacterial PLFAs for living bacterial biomass. Laccase activity was absent in PII-end compost during the incubation period in the absence of *A. bisporus*, while chitin and C18:2ω6 content increased by 26% and dropped by 50%, respectively. In contrast, laccase activity and C18:2ω6 levels increased during the first 19 days of *A. bisporus* colonization, while chitin content already reached its maximum at day 13. These data confirm that laccase is a good marker for vegetative growth of *A. bisporus* in compost as reported previously (Wood 1979). However, its activity (Wood and Goodenough 1977) and transcript levels (Ohga et al. 1999) drop during mushroom formation and therefore laccase can only be used as a biomarker during PIII.

Standard curves of chitin and PLFA content in pure mycelium of *A. bisporus* were used to estimate fungal biomass in compost. This approach assumes that chitin and PLFA content is identical between species and culture conditions, which is not necessarily the case. For instance, C18:2ω6 content ranges from 1.4 to 22.9 nmol mg^−1^ between fungal species with basidiomycetes containing 45–57 mol% and zygomycetes between 12 and 22 mol% (Klamer and Bååth 2004). Using these numbers, 25 and 400 mg g^−1^ represent the extremes of fungal biomass in PII-end compost. Notably, *A. bisporus* contains 40 nmol mg^−1^ C18:2ω6 and this number was used to estimate fungal biomass in this study. The use of the high C18:2ω6 content of *A. bisporus* to calculate fungal biomass does not impact relative changes found in this study but is expected to underestimate the fungal biomass in compost 2–4 fold in the absence of *A. bisporus.* Chitin levels can vary depending on culture age and growth conditions (Sharma et al. 1977). In the case of *A. bisporus*, however, chitin content did not change in time relative to fungal dry weight in malt extract (Matcham et al. 1985). Moreover, *A. bisporus* becomes the dominant fungal species after its inoculation. Fungal biomass in *A. bisporus* inoculated compost based on chitin and PLFA was calculated to amount 68 and 29 mg g^−1^ compost after 26 days. This indicates that more than half of the fungal biomass is dead. The 50% decrease in living fungal biomass in the absence of *A. bisporus* shows that the applied growth conditions negatively impact the established fungal community in PII compost. At the same time, the increase in chitin content under this condition suggests that part of the fungal community continues to grow, albeit slower than the death rate.

Relative abundance of bacterial PLFAs changed during the 26-day-period. This shows that the bacterial community is influenced by the vegetative growth of *A. bisporus* and that (selected) bacteria divide and thus produce biomass. The relative increase of the Gram positive associated markers a15:0 and a17:0 and the relative decrease of Gram negative associated markers cy17:0 and cy19:0 indicates that Gram negative bacteria are more suppressed by *A. bisporus* than Gram positive bacteria. Previously, it was calculated that the microbial biomass consumption would contribute <10% of the mushroom dry weight biomass (Sparling et al. 1982). This was based on colony forming units in PII-end compost extracts and by direct counting. It did not include bacterial biomass formation in PIII and therefore bacterial consumption could be much higher. The fact that bacterial biomass was relatively stable in the absence of *A. bisporus,* while it decreased about fourfold in the 26-day-period in the presence of *A. bisporus* supports the hypothesis that this mushroom forming fungus feeds on bacteria. This is also suggested from the fact that growth of *A. bisporus* is strongly reduced when it is exposed to sterilized compost (not shown). Based on a conversion factor from the literature the PII-end compost contained 8.9 mg C bacterial biomass g^−1^ compost (Frostegård and Bååth 1996; Tsiafouli et al. 2015). When assuming a C content of 50% in dry material of bacteria (Bratbak and Dundas 1984) a decrease in living bacterial biomass from 17.7 to 4.7 mg g^−1^ compost occurred during the 26 day period of compost colonization by *A. bisporus*. The increase in fungal biomass from 14 to 30 mg g^−1^ in the same period and the fact that bacterial biomass production in PIII is not taken into account suggests that the *A. bisporus* diet may contain bacteria as its main course. In this view, *A. bisporus* would secrete the enzymes that degrade lignocellulose. The bacteria would use the degradation products as nutrients, after which *A. bisporus* feeds on the bacterial biomass. This strategy would alleviate the deficiency of *A. bisporus* to produce molecules such as vitamins.

## Additional file

**Additional file 1.** Additional table and figure.

## Abbreviations

CAM: compost agar medium
GlcNAc: *N*-Acetylglucosamine
MEA: malt extract agar
PI: phase I
PII: phase II
PIII: phase III
PIV: phase IV
PC: polycarbonate
PLFA: phospholipid-derived fatty acid
U: units
