# Supplementary material for: Microbial biomass in compost during colonization of Agaricus bisporus
Source: AMB Express. 2017 Jan 3;7:12. doi: 10.1186/s13568-016-0304-y (PMC5209305; doi:10.1186/s13568-016-0304-y)
Supplement: Supplementary file 1 — Additional file 1. Additional table and figure. [file 13568_2016_304_MOESM1_ESM.pdf]

AMB Express

Microbial biomass in compost during colonization of Agaricus bisporus

Aurin M. Vos[1], Amber Heijboer[2], Henricus T.S. Boschker[3], Barbara Bonnet[4], Luis G. Lugones[1], Han A. B. Wösten[1]

[1]Microbiology, Department of Biology, Utrecht University, Padualaan 8, 3584 CH Utrecht, The Netherlands; [2]Biometris, Wageningen University, Droevendaalsesteeg 1, 6708 PB Wageningen, The Netherlands; [3]Koninklijk Nederlands Instituut voor Onderzoek der Zee, Korringaweg 7, 4401 NT Yerseke, The Netherlands; [4]Somycel S.A-Sylvan, Z.I. Sud, Route de Tours, 37130 Langeais, France

Corresponding author

Prof. dr. Han A.B. Wösten

Department of Microbiology, Utrecht University

Padualaan 8, 3584 CH Utrecht

The Netherlands

Telephone: 31 30 2533448

Fax: 31 30 2513655

E-mail: h.a.b.wosten@uu.nl

Table S1: Bacterial PLFA markers used in this study (adapted from Ruess and Chamberlain 2010; Heijboer et al. 2016).

PLFA markers	Fatty acid type	Predominant origin	References
i15:0, a15:0, i16:0, a17:0	Ise/anteiso methyl-branched	G+ bacteria	Zelles (1997, 1999)
cy17:0, cy19:0	Cyclopropyl ring	G- bacteria	Zelles (1997, 1999)
15:0	Straight-chain saturated < 19	Non-specific	
10Me16:0	10-methyl-branched	Sulphate reducing bacteria and/or Actinomycetes	Dowling et al. (1986) & Kerger et al. (1986)
18:1ω7	Double bond C7	Bacteria	Zelles (1999)
16:1ω9	Double bond C9	Non-specific	
18:2ω6	Poly-unsaturated ω6 family	Sapotrophic fungi	Frostegard & Baath (1996) & Zelles (1999)

Dowling NJ, Widdel F, White DC (1986) Phospholipid ester-linked fatty acid biomarkers of acetate-oxidizing sulphate-reducers and other sulphide-forming bacteria. Microbiology 132:1815-1825.

Frostegård Å, Bååth E (1996) The use of phospholipid fatty acid analysis to estimate bacterial and fungal biomass in soil. Biol Fertility Soils 22:59-65.

Heijboer A, ten Berge HF, de Ruiter PC, Jørgensen HB, Kowalchuk GA, Bloem J (2016) Plant biomass, soil microbial community structure and nitrogen cycling under different organic amendment regimes; a 15 N tracer-based approach. Appl Soil Ecol 107:251-260.

Kerger BD, Nichols PD, Antworth CP, Sand W, Bock E, Cox JC, Langworthy A, White DC (1986) Signature fatty acids in the polar lipids of acid-producing *Thiobacillus* spp.: Methoxy, cyclopropyl, alpha-hydroxy-cyclopropyl and branched and normal monoenoic fatty acids. FEMS Microbiol Lett 38:67-77.

Ruess L, Chamberlain PM (2010) The fat that matters: soil food web analysis using fatty acids and their carbon stable isotope signature. Soil Biol Biochem 42:1898-1910.

Zelles L, Palojärvi A, Kandeler E, Von Lützow M, Winter K, Bai QY (1997) Changes in soil microbial properties and phospholipid fatty acid fractions after chloroform fumigation. Soil Biol Biochem 29:1325-1336.

Zelles L (1999) Fatty acid patterns of phospholipids and lipopolysaccharides in the characterization of microbial communities in soil: a review. Biol Fertility Soils 29:111-129.

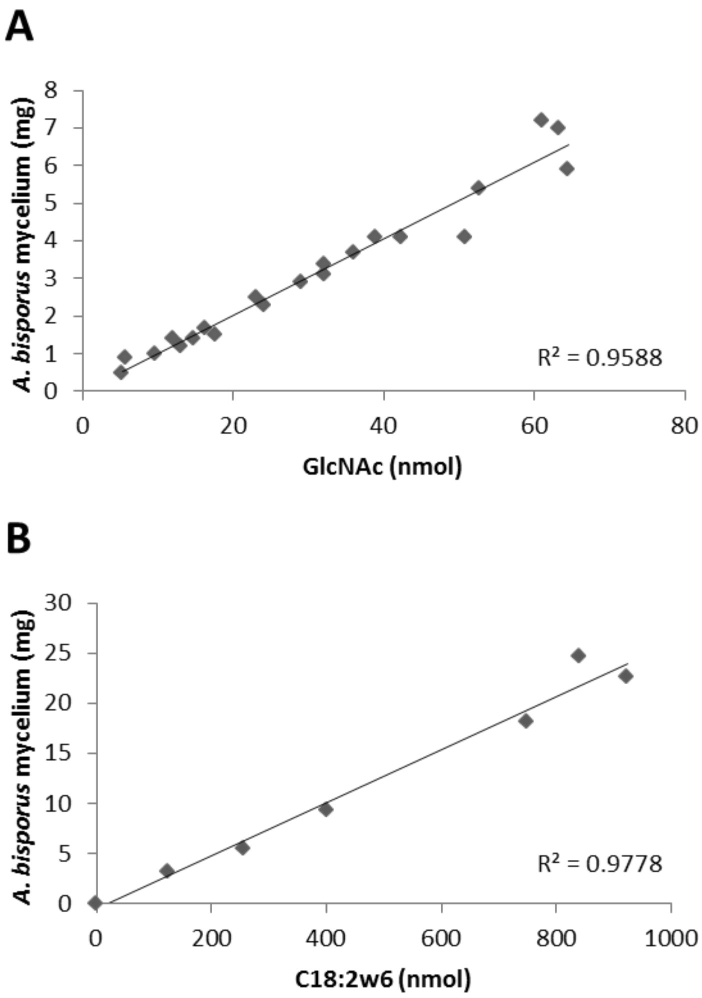

Figure S1: N-acetylglucosamine (GlcNAc) (A) and PLFA marker C18:2ω6 (B) content in pure *A. bisporus* mycelium.
